# Structure-Based Development of SARS-CoV-2 Spike Interactors

**DOI:** 10.3390/ijms23105601

**Published:** 2022-05-17

**Authors:** Flavia Squeglia, Maria Romano, Luciana Esposito, Giovanni Barra, Pietro Campiglia, Marina Sala, Maria Carmina Scala, Alessia Ruggiero, Rita Berisio

**Affiliations:** 1 Institute of Biostructures and Bioimaging, IBB, CNR, 80131 Napoli, Italy; flavia.squeglia@cnr.it (F.S.); maria.romano@cnr.it (M.R.); luciana.esposito@cnr.it (L.E.); giovanni.barra@unicampania.it (G.B.); 2 Department of Pharmacy, University of Salerno, 84084 Fisciano, Italy; pcampiglia@unisa.it (P.C.); msala@unisa.it (M.S.); mscala@unisa.it (M.C.S.)

**Keywords:** SARS-CoV-2, COVID-19, viral entry, spike protein, protein structure, infectious disease

## Abstract

Coronaviruses, including SARS-CoV-2 (the etiological agent of the current COVID-19 pandemic), rely on the surface spike glycoprotein to access the host cells, mainly through the interaction of their receptor-binding domain (RBD) with the human angiotensin-converting enzyme 2 (ACE2). Therefore, molecular entities able to interfere with the binding of the SARS-CoV-2 spike protein to ACE2 have great potential to inhibit viral entry. Starting from the available structural data on the interaction between SARS-CoV-2 spike protein and the host ACE2 receptor, we engineered a set of soluble and stable spike interactors, here denoted as S-plugs. Starting from the prototype S-plug, we adopted a computational approach by combining stability prediction, associated to single-point mutations, with molecular dynamics to enhance both S-plug thermostability and binding affinity to the spike protein. The best developed molecule, S-plug3, possesses a highly stable α-helical con-formation (with melting temperature Tm of 54 °C) and can interact with the spike RBD and S1 domains with similar low nanomolar affinities. Importantly, S-plug3 exposes the spike RBD to almost the same interface as the human ACE2 receptor, aimed at the recognition of all ACE2-accessing coronaviruses. Consistently, S-plug3 preserves a low nanomolar dissociation constant with the delta B.1.617.2 variant of SARS-CoV-2 spike protein (K_D_ = 29.2 ± 0.6 nM). Taken together, we provide valid starting data for the development of therapeutical and diagnostic tools against coronaviruses accessing through ACE2.

## 1. Introduction

The coronavirus SARS-CoV-2 has spread widely and rapidly since it was first identified in Wuhan, China in December 2019 [1,2,3]. Its associated disease, COVID-19, causes severe respiratory difficulties, with aged patients at higher risk of mortality [1]. Given the dramatic public health emergency and the growing number of variants that have emerged so far, there is a strong and urgent need for new antiviral agents to block human-to-human transmission and to treat infected patients. 

Like other coronaviruses, SARS-CoV-2 makes use of a densely glycosylated spike protein to gain access into host cells [4,5]. The spike protein forms homo-trimers protruding from the viral envelope and binds with high affinity to the host receptor ACE2 (angiotensin-converting enzyme 2), mainly expressed by epithelial cells of the respiratory tract [6]. After attachment, the human transmembrane protease serine 2 (TMPRSS2) cleaves and activates the spike protein, thus allowing SARS-CoV-2 to enter the host cells [7]. Compared to SARS-CoV, an additional protease, possibly furin, is likely involved in priming the SARS-CoV-2, because the spike protein of SARS-CoV-2 contains four redundant furin cut Pro-Arg-Arg-Ala motifs that are absent in SARS-CoV [6]. Once inside the cell, the infecting RNA acts as a messenger RNA, which is translated by host ribosomes to produce components necessary to assemble the new viral particles [8,9].

The spike protein contains two subunits, S1 and S2. Of these, S1 comprises the receptor-binding domain (RBD), which is responsible for recognising and binding the cell surface receptor ACE2 [5]. As it is essential for infection, the spike protein has been exploited as a target for antibodies and vaccines [10]. It is the principal target of the neutralising antibodies generated following infection by SARS-CoV-2 [11,12]. Consistently, it is the antigen of both mRNA and adenovirus-based vaccines licensed for use and others awaiting regulatory approval [13]. As SARS-CoV-2 continues to spread and cause diseases, emerging variants are being identified around the globe. As a consequence, there is emerging evidence of reduced neutralisation of some SARS-CoV-2 variants [13]. In addition, regarding the effectiveness of neutralising antibodies, their therapeutic and diagnostic use has several limitations associated with their large molecular mass, which often causes poor tissue penetration [14,15]. These features prompted researchers to develop smaller and less expensive protein scaffolds as alternatives to antibodies [15]. In addition to this, the large number of spike mutations so far observed [16], and the potential impact of mutations on the antigenicity of the spike protein, still prompt a strong interest in identifying spike-interacting molecules with high resemblance to the ACE2 receptor as a tool to target all variants that are able access the host through the ACE2 receptor, both for therapeutic and diagnostic use. 

Based on the structural information available on the complex between the spike protein and the ACE2 receptor [17], we developed high-affinity binders to the spike with the property to share the same interacting interface with ACE2. The first developed molecule, S-plug, was highly soluble and displayed nanomolar binding affinity with the spike protein of SARS-CoV-2. By combining mutational stability prediction analysis with molecular dynamics (MD) simulations, we identified possible mutations to enhance S-plug thermostability. The resulting proteins, S-plug2 and S-plug3, displayed significantly enhanced melting temperatures and low nanomolar dissociation constant (K_D_) to the spike protein. 

## 2. Results 

### 2.1. Design of S-plug and Thermostabilised S-Plugs

**Figure 1:**
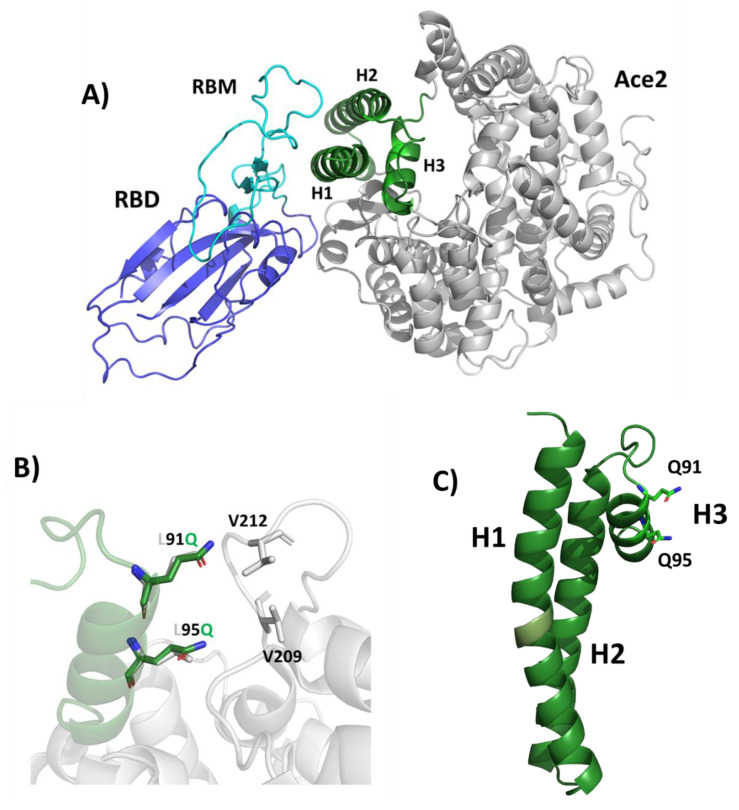
Design of S-plug based on spike RBD-ACE2 complex structure. (**A**) Cartoon representation of the complex between the RBD of SARS-CoV-2 spike protein (blue/cyan) and the human ACE2 receptor (grey/green). The receptor-binding motif (RBM) is drawn in cyan. The green portion of the ACE2 domain, including helices H1, H2 and H3, is drawn in green. (**B**) Cartoon representations showing the superposition of S-plug (green) on ACE2 (grey). Leu91 and Leu95, involved in hydrophobic interactions with V209 and V212 in the ACE2 structure, were mutated to glutamine residues in S-plug. (**C**) The structure of S-plug, mutated residues are shown in stick. Figures were generated with Pymol [18].

An analysis of the structure of SARS-CoV-2 complex with ACE2, reveals that most hydrogen bonds between the spike protein and ACE2 involve the helix H1 (residues 20–52) and the C-terminal part of helix H2 (residues 56–82) of ACE2 [17]. Starting from the region H1-H2 of ACE2 as a basic scaffold for the design of a spike interactor, we included several mutations to increase its stability and solubility, with the aim to compensate for the missing interactions that are mediated by residues outside helices H1 and H2. To stabilise this scaffold, we included an extra helix, H3 (residues 91–101) which naturally caps helices H1 and H2 in the ACE2 structure, through a cluster of hydrophobic interactions involving Val92, Leu97, and Leu100 of H3; Phe28, Leu29, and Phe32 of H1; and Ala80 of H2, holding together the two helices, H1 and H2 (Figure 1). We further engineered this variant by including two mutations, Leu91 to Gln and Leu95 to Gln, to improve protein solubility by replacing hydrophobic with hydrophilic residues, and hamper aggregation in solution. In ACE2, these two residues are located on the opposite side of the S-binding region and form hydrophobic interactions with the core of ACE2 to stabilise it (Figure 1B,C). Their mutation to glutamine residues was also motivated by the characteristics of glutamine to further stabilise α-helix structures. 

An important issue in peptide or small protein design is thermal stability, because peptides are usually not folded in solution. Therefore, our further goal was to identify thermo-stabilising mutations, located in the region that does not interact with the spike protein, to preserve its binding. Using Rosetta mutation analysis [19], we identified a series of predicted thermo-stabilising mutations, acting either by increasing the buried hydrophobic surface area, or by increasing the quality of side-chain packing. Of the identified mutations, 10 were located at the interface between S-plug and RBD and were not applied to prevent destabilisation of the S-plug–RBD complex. To validate the effect of the mutations identified as stabilising (but not disruptive to the complex) on the structural integrity and flexibility of the proteins, we combined this strategy with MD simulations of S-plug and its mutated form, S-plug2 (Table 1). 

### 2.2. MD Simulations

**Figure 2:**
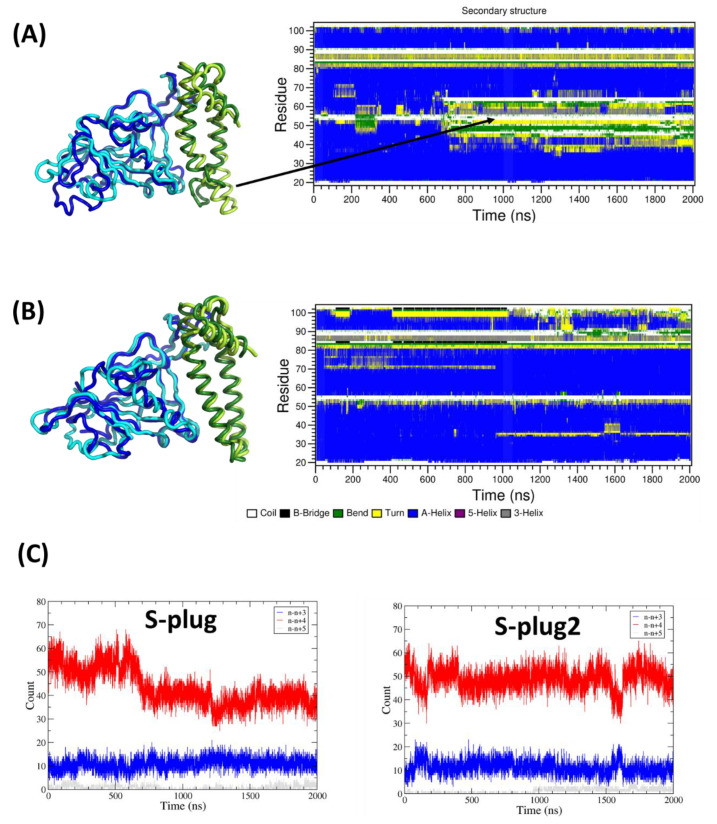
Molecular dynamics (MD) studies. (**A**,**B**) Secondary structure time evolution of S-plug and S-plug2, respectively, in the MD simulations complexes; best superimposition of the starting and average structures of the trajectories are reported on the left panels; RBD is shown in cyan/blue, S-plugs in yellow/green (starting/average); average structures (on Cα atoms) are computed over the entire trajectories. (**C**) Evolution of the number of hydrogen bonds in helical conformation in S-plug (left) and S-plug2 (right).

Two independent 2 µs MD simulations computations were performed, starting from complexes of the RBD of the spike protein (residues 336-516) with either S-plug or S-plug2. The evaluation of root-mean-square deviations (RMSD) (calculated on the protein Cα atoms) between the starting models and the trajectory structures shows limited conformational variations of the entire system, with RMSD values close to four Å in both simulations (Figure S1A,B). However, when focusing on the S-plug chains, the analysis of secondary structure (SS) shows a clear loss of α-helical conformation for the C-terminal part of H1 (residues 40–51) and the N-terminal of H2 (residues 57–65) of S-plug in the MD simulation of RBD–S-plug (Figure 2A). By contrast, stable SS evolution characterises the MD simulation of the RBD–S-plug2 complex (Figure 2B). Consistently, the number of hydrogen bonds stabilising α-helices (n + 4) of S-plugs strongly decreases throughout the MD simulation trajectory of RBD–S-plug, whereas it remains constant in the RBD–S-plug2 MD simulation (Figure 2C). Additionally, the RMSD between the starting models and the trajectory structures, computed on the 40-65 region, shows a remarkable increase in the RBD–S-plug MD trajectory (from one to five Å), whereas it remains unaltered (close to one Å) in the RBD–S-plug2 MD trajectory (Figure S1C,D). Importantly, the region 40-65 is not predicted to affect the binding affinity of spike interactors because the corresponding region of ACE2 is not involved in spike binding. Therefore, better organisation of this region in an α-helical arrangement in S-plug2 is likely to strongly contribute to protein stability without decreasing its binding affinity to the spike protein. 

### 2.3. S-Plugs Folding and Thermostability

**Figure 3:**
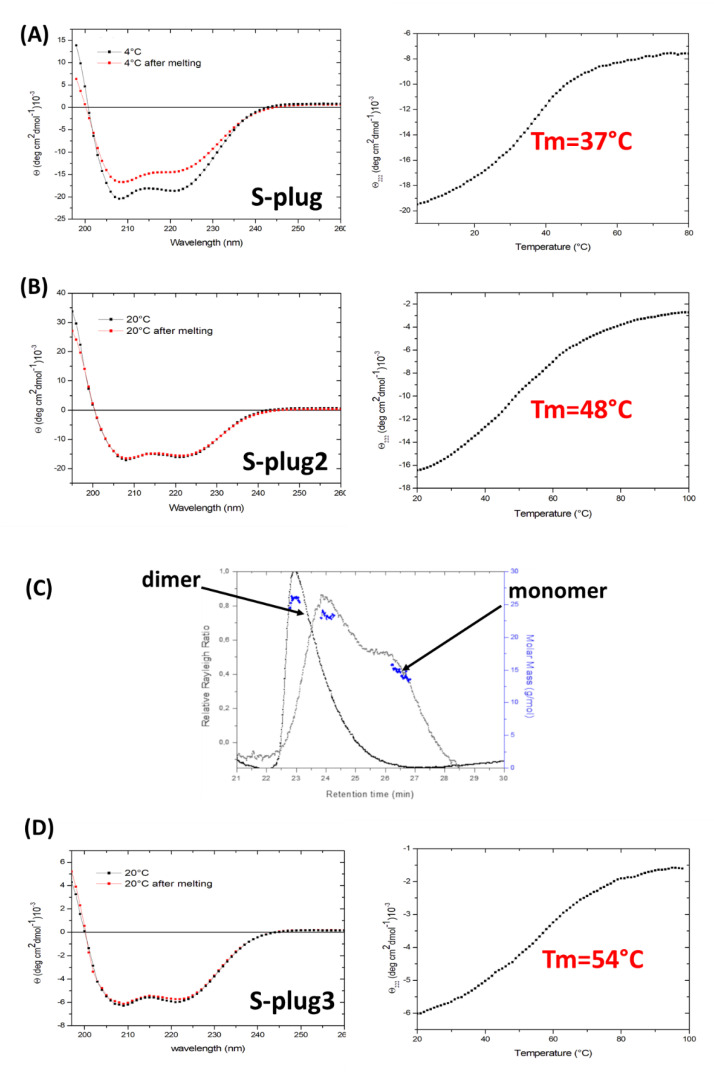
Thermostability and light scattering studies. (**A**,**B**,**D**) Far-UV CD spectra (left) and thermal denaturation curves monitored at 222 nm (right) for S-plugs. All spectra were measured at 0.2 mg mL-1 in in 20 mM sodium phosphate buffer (pH 7.4). The panel (**C**) reports the analytical SEC-LS of S-plug (grey) and S-plug2 (black). Relative Rayleigh ratios (left scale) and derived molar masses (right scale, blue) are plotted versus elution time.

Designed miniproteins were successfully over-expressed in *E. coli*, resulting in high yields of pure proteins. Far-UV CD spectroscopy spectra of S-plug and S-plug2 are typical of well-structured folds with high α-helical content, with typical minima at 208 and 222 nm (Figure 3). Spectra also show that folding is fully reversible, with the CD spectrum after refolding fully superimposable to that recorded at 20 °C (Figure 3A). To investigate the heat-induced changes in the protein secondary structure, thermal unfolding curves were recorded by following the CD signal at 222 nm as a function of temperature, using a 1 °C/min heating rate. Thermal unfolding curves of S-plug and S-plug2 exhibit the characteristic sigmoidal profile expected of two-state systems (Figure 3A,B). Consistent with MD computations, S-plug2 displays a thermo-stabilised structure, with an increase in the melting temperature (Tm) from 37 °C of S-plug to 48 °C (Figure 3A,B). 

**Figure 4:**
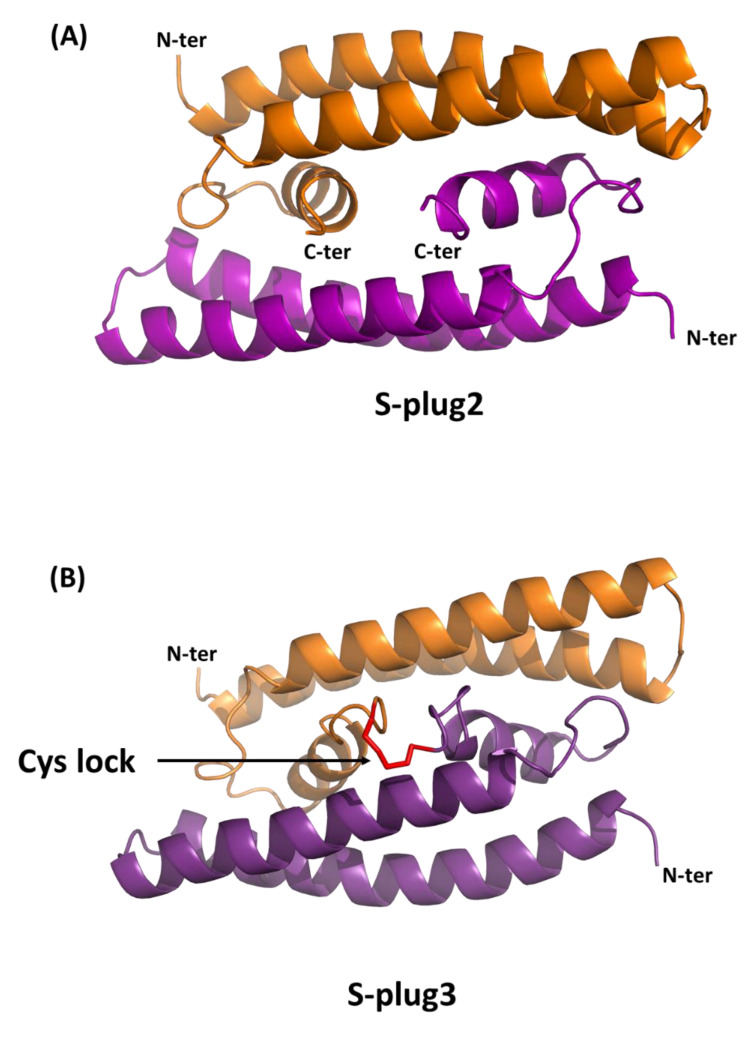
Cartoon representations of S-plug2 (**A**) and S-plug3 (**B**) models obtained using Rosetta docking procedures [19]. The C-terminal arm (PGGC) inserted in S-plug3 to induce a disulphide bond (**B**) is drawn in red ribbon representation.

Analysis of the oligomeric states of S-plug and S-plug2 shows that S-plug exists in solution in equilibrium between a monomeric and dimeric state, whereas S-plug2 is mostly dimeric (Figure 3C). This result indicates that part of the observed protein stabilisation achieved is due to the stabilisation of the dimeric state in S-plug2. Using the docking procedure implemented in Rosetta software, [19] we computed a three-dimensional model of the S-plug2 dimer (Figure 4). As shown in Figure 4, the S-plug2 dimer leaves the spike-interacting regions on the two opposite sides of the molecule, as two molecules are placed back-to-back. In this arrangement, the C-termini of the two monomers are in close proximity. Therefore, we further modified S-plug2 sequence to add a C-terminal arm ending with a cysteine residue (with sequence PPGC, Table 1), to induce a covalent bond formation (Figure 4). The miniprotein with the resulting sequence, S-plug3, was over-expressed in *E. coli* and checked for its dimeric state by mass spectrometry. As reported in Figure 3D, CD spectroscopy data show that the cysteine-lock induced in S-plug3 further stabilised the molecule, as its denaturation temperature further increased from 48 to 54 °C. All developed miniproteins were assayed for their ability to interact with the spike protein. 

### 2.4. Binding Analyses

Real-time binding assays were performed to assess binding kinetics and affinity between developed miniproteins and either the RBD or the S1 domain of the spike protein from SARS-CoV-2, using Biacore T200 (Cytiva, Uppsala, Sweden). The spike RBD-His was stably captured at the surface of the CM5 sensor chip using an anti-histidine antibody that had been covalently bound to the surface, as recommended by the manufacturer. The SARS-CoV-2 spike protein (S1) was immobilised on a CM5 sensorchip using standard amine-coupling protocols (see methods). Following immobilisations, protein solutions (S-plug, S-plug2, S-plug3) were injected at various concentrations (from 0.005 to 5µM), using a flow rate of 30 μL/min for 120 s (association phase), and then the buffer alone for 600 s (dissociation phase). Equilibrium dissociation constant (K_D_) values were derived from the ratio between kinetic dissociation (kd) and association (ka) constants, obtained by fitting data from all injections at different concentrations of each compound, using the simple 1:1 Langmuir binding fit model of the BIAevaluation software (version 2.0.2).

**Figure 5:**
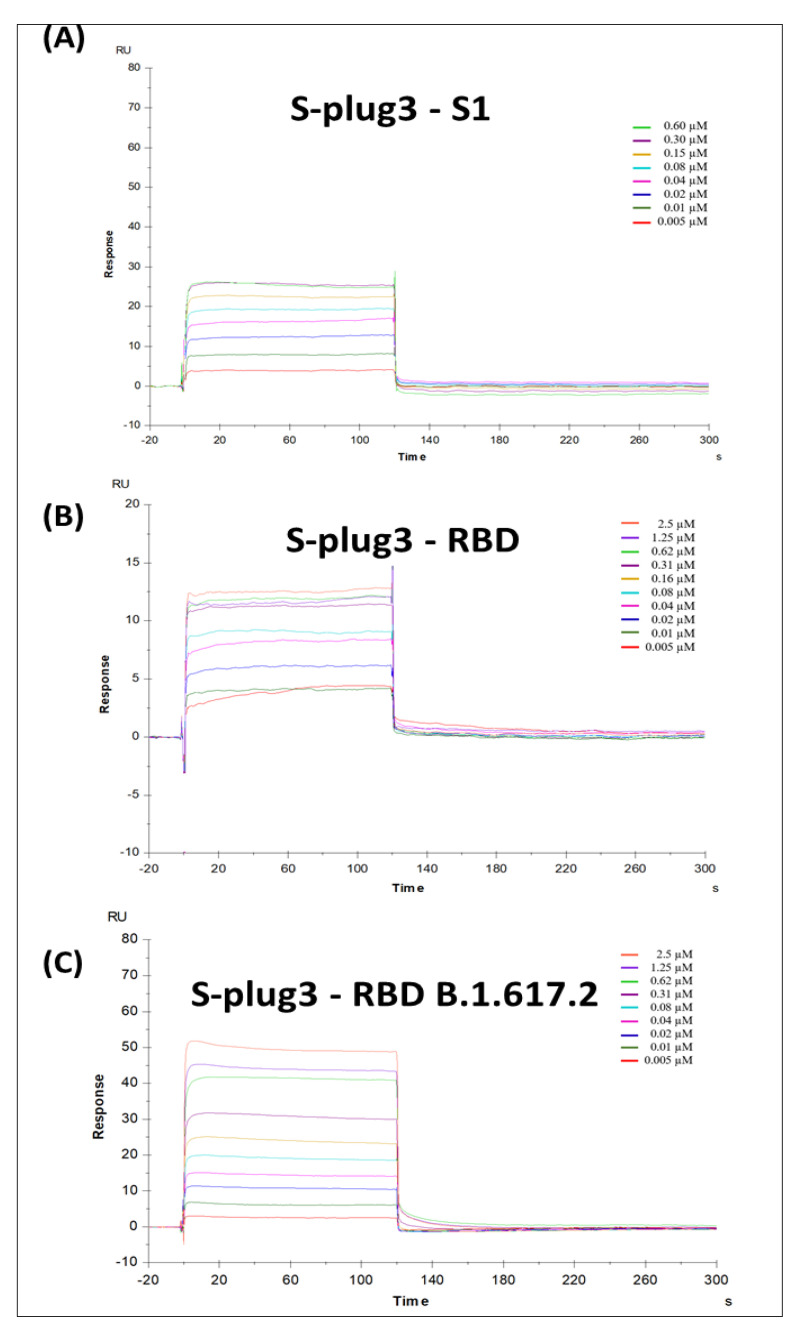
Surface plasmon resonance (SPR) analysis. Sensorgrams to evaluate the binding affinity between S-plug3 and (**A**) spike S1 domain, (**B**) spike RBD, (**C**) spike RBD B.1.617.2 (delta) variant.

Results showed strong binding of S-plug to either RBD or S1, in the range 50–200 nM. However, low reproducibility characterised these experiments, depending on the preparation and time of storage. This behaviour is likely due to the conformational variability we observed for this construct, both in terms of secondary structure stability and of oligomerisation state (Figure 2 and Figure 3). Highly reproducible binding was observed for all other miniproteins. In particular, SPR analysis shows that S-plug2 binds to either the spike RBD or the S1 domain with elevated affinity, with K_D_ values of 61.7 ± 2.3 nM and 71.7 ± 1.8 nM, respectively (Table 2, Figure S3). The strongest binding characterises S-plug3, with K_D_ to RBD of 39.5 ± 1.1 nM and to S1 of 31.7 ± 1.8 nM (Table 2, Figure 5). Given this result, we also tested the binding of S-plug3 to the delta variant of spike RBD (B.1.617.2, carrying the mutations L452R and T478K), one of the deadliest variants so far reported [20]. Results show strong binding of S-plug3, with K_D_ of 29.2 ± 0.6 nM (Figure 5). We also measured the affinity of spike S1 domain to the ACE2 receptor as a control experiment, which provided a dissociation constant K_D_ of 51.9 ±1.4 nM (Table 2 and Figure S2).

## 3. Discussion

Several structures of the spike protein in different functional states and of complexes between the spike protein and the ACE2 receptor have recently become available [5,17,21,22,23,24]. In the prefusion state, the RBDs of the spike protein alternate between open ‘up’ and closed ‘down’ conformations. Consequently, the receptor-binding site, which can bind to human angiotensin-converting enzyme 2 ACE2, is transiently exposed in the ‘up’ conformation [25]. Many neutralising antibodies are directed against the RBD, with the highest neutralising activity associated with antibodies binding the RBD in the down state [11,12,26]. However, severe limitations are associated with the use of antibodies as therapeutics due to their typical suboptimal pharmacokinetics [27]. Their use is also limited for diagnostic purposes, due to the elevated costs of production. Miniaturisation represents a major direction in the development of rationally designed, less expensive protein scaffolds as alternatives to antibodies [15,27].

Based on the available structural information of the spike–ACE2 complex, we developed miniproteins which embed key spike-interacting residues in the ACE2 receptor. After the development of our prototype miniprotein spike binder, S-plug [28], one study reported the development of other miniproteins binding to the spike with high affinity; the best ranking showed low nanomolar binding affinity and was based on large-scale de novo design [29]. We started from our prototype S-plug [28], containing H1-H2 and H3 helices of ACE2, with mutations on the H3 helix (L91Q and L95Q) to improve its solubility (Figure 1). Then, our protein-engineering goal was to identify thermo-stabilising mutations located in regions that do not interact with the spike protein, to preserve its binding mode. The rationale of this study is to generate spike interactors that resemble the human ACE2 receptor, as we expect that such molecules would recognise all known coronaviruses accessing the host through this receptor, such as SARS-CoV, HCoV-NL63, as well as other coronaviruses that may emerge in the future. 

**Figure 6:**
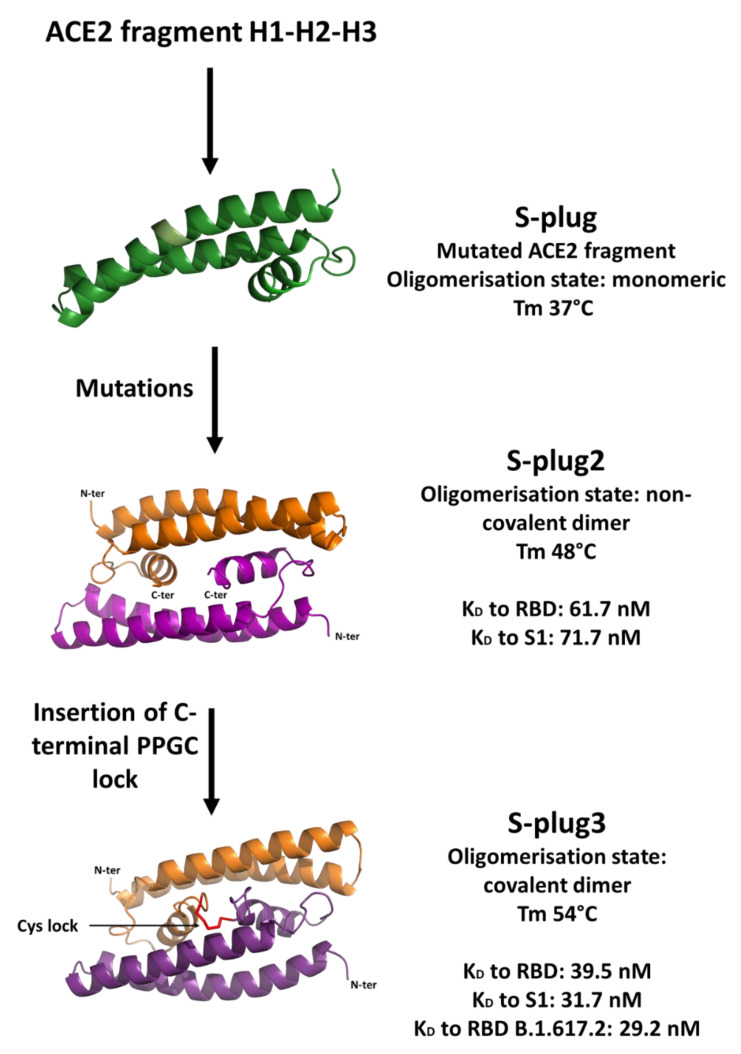
A sketch of the S-plugs’ design strategy with a summary of their thermal stabilities and binding affinities to the spike RBD and S1 domain.

**Figure 7:**
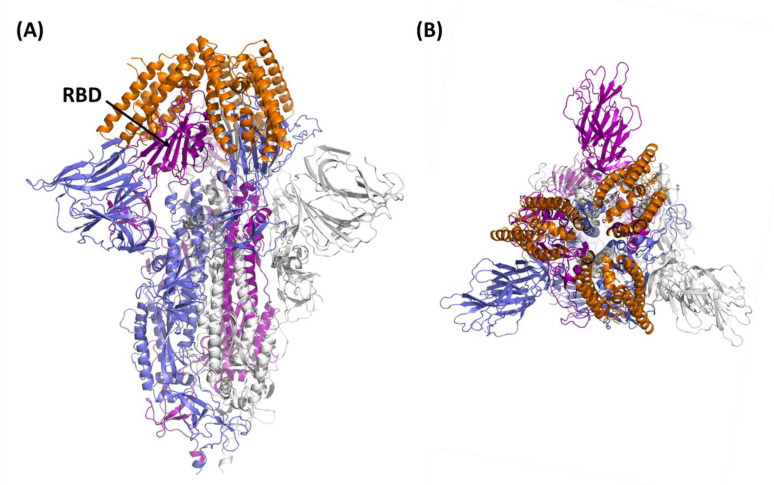
Cartoon representation of the spike trimer (closed conformation) in complex with S-plug3. The positions of S-plug3 molecules (orange) on the spike protein were determined upon superposition of RBD of our minimised S-plug3–RBD complex on the RBD of the entire S protein (pdb code 6m0j). (**A**,**B**) panels report side and top views, respectively.

Flexible regions are typically considered “hot spots” for engineering protein thermostability. Using MD simulations, we predicted that the introduced mutations induce a higher stability of α-helical and loop regions (residues 40–65), which are not involved in spike binding. Consistently, we recombinantly produced the resulting miniprotein, S-plug2, and showed that it presents a highly stable α-helical conformation in solution, with a Tm of 48 °C, 11 °C higher than the original S-plug (Tm 37 °C). Additionally, S-plug2 displays a low nanomolar dissociation constant K_D_, determined here using surface plasmon resonance, to both the RBD of glycosylated S protein and to the entire S1 region of SARS-CoV-2 (Figure 5 and Figure 6). Based on docking analysis of S-plug2, which presents a dimeric organisation in solution, we further improved it by adding a C-terminal arm ending with a cysteine residue to induce a cysteine lock. This protein, which we named S-plug3, is more stable than S-plug2, with a Tm of 54 °C, and displays enhanced binding affinities with both the spike S1 domain and RBD (Figure 6). This affinity is comparable to those hitherto measured by SPR with the ACE2 receptor (Table S2) [5,17,24]. Additionally, molecular dimensions of S-plug3 are compatible to full saturation of the RBDs in the spike trimer (Figure 7).

In conclusion, we designed and developed highly stable spike interactors, sharing with the ACE2 receptor a highly resemblant spike interface, aimed at the recognition of all coronaviruses accessing the host through the ACE2 receptor. Consistently, we show that our best performing miniprotein, S-plug3, binds to the deadliest delta variant of spike RBD with a dissociation constant K_D_ of 29.2 (±0.6) nM. The most immediate application of these molecules is for diagnostic purposes, as the validation of their therapeutic properties still awaits a long experimental assessment. Taken together, data reported here provide a protocol for the design and development of stable miniproteins that mimic larger host receptors and valid starting tools for the development of therapeutic and/or diagnostic measures against ACE2-accessing coronaviruses.

## 4. Experimental Methods

### 4.1. Computational Design of S-Plug and the Thermo-Stabilised S-Plugs

The crystal structure of the complex between ACE2 and the RBD of the S protein from SARS-CoV-2 (PDB code 6m0j) was used as a starting model to design the spike interactors [17]. Interactions between the spike protein and ACE2 were computed to identify the smallest region of the ACE2 receptor able to bind the S-protein. This region, including residues 19-103 of the ACE2 receptor, was suitably mutated to enhance protein solubility (Table 1). Mutations in the basic scaffold were generated using the software Coot [30] and models were energy minimised using the GROMACS package to check the structural effect of mutation [31].

To design spike interactors with increased thermal stability, we used a combined computational approach including (i) structure-guided rational design methods, using Rosetta [32] with (ii) molecular dynamics approaches, using GROMACS [31]. Starting from the model of S-plug, generated as explained above, Rosetta software was used to predict the relative change in folding free energy of residues in flexible regions due to point mutations [32]. Key residues for interactions with RBD were left unaltered. Inserted mutations were selected to either increase buried hydrophobic surface area or increase the quality of side-chain packing. To predict structural stability changes of the spike interactors, we adopted MD dynamics, starting from either S-plug or the modified variant (S-plug2) in complex with spike RBD. 

### 4.2. Molecular Dynamics

Simulations were performed by the GROMACS package [31], using protocols already tested for other protein systems [33,34,35]. Starting models were obtained as described above. For each starting model, 2 µs MD simulations were carried out using the Amber99sb all-atom force field [36,37] with tip4pew water model. All simulations were run in the isobaric-isothermal (NPT) ensemble at 300 K, using periodic boundary condition. 

The starting models were solvated in a dodecahedron box with minimal distance of the model to the box wall of 11 Å. Sodium or chloride ions were added by replacing water molecules to neutralise the overall charge. Details are listed in Table S1. Before starting MD simulations, the systems were subjected to energy minimisation, then the solvent was equilibrated first in NVT and then in NPT ensemble for 100 ps, during which the protein atoms were restrained to the energy-minimised initial coordinates. Subsequently, the thermalisation process consisted of a sequence of 100-ps runs where the temperature was increased from 50 K to 300 K in 50 K steps. At this stage, the Berendsen method for pressure control was used with a coupling constant τ_p_ = 1.0 ps, whereas the temperature was kept constant at 300 K by the V-rescale method with a τ_t_ = 0.1 ps. For the production run, the pressure coupling was employed using the Parrinello–Rahman barostat with τ_p_ = 2.0 ps. The integration time step was 0.002 ps and LINCS was the constraint algorithm used [38]. The electrostatic interactions were treated using the particle-mesh Ewald (PME) method with a real-space cutoff of 10 Å [39]. Lennard-Jones interactions were truncated at 10 Å [40]. All the trajectories were analysed using GROMACS routines. Structural properties, such as root mean-square deviation (RMSD), root mean-square fluctuation (RMSF), and secondary structure (SS), were calculated with GROMACS or Visual Molecular Dynamics (VMD) [41] standard analysis tools.

### 4.3. Protein Expression and Purification

The genes encoding the S-plug and S-plug2 proteins were synthesised by GeneArt® (ThermoFisher), and subsequently subcloned in pETM-11 expression vector (EMBL, Germany) between the NcoI and XhoI restriction sites. The S-plug3 variant was produced by polymerase chain reaction (PCR), by introducing a spacer consisting of three amino acids and terminating a cysteine residue (-PPGC) at the protein C-terminal end, using the following oligonucleotides: Forward 5′-CATGCCATGGCGAGCGCGATGG-3′; Reverse 5′-CCGCTCGAGTTAGCAGCCCGGCGGGCTGCTCGCGGTTTTTTCC-3′. The PCR product was cloned in pETM-11 expression vector. All proteins were expressed with a TEV-cleavable N-terminal histidine tag. The expression levels were optimised after carrying out small-scale expression screening using different strains of *E. coli*. The recombinant proteins were successfully over-expressed in *E. coli* BL21(DE3) cells. Briefly, an overnight starting culture of 10 mL was prepared for the growth with 1L of Luria Bertani (LB) medium containing 50 µg/mL kanamycin, which was then induced by adding 0.8 mM of IPTG at 18 °C for approximately 16 h. For protein purification, a similar procedure was adopted. The harvested bacterial cells were resuspended in the binding buffer (50 mM Tris-HCl, 300 mM NaCl, 5% (*v/v*) glycerol and 10 mM imidazole, pH 7.8) containing a complete protease inhibitor cocktail (Roche Diagnostics, Mannheim, Germany), and then the cell suspension was lysed by sonication. The lysate was cleared by centrifugation at 18,000× *g* at 4 °C and the supernatant was loaded onto 5 mL of Ni–NTA resin (Qiagen, Milan, Italy) equilibrated with the binding buffer. After washing with ten volumes of binding buffer, the protein was eluted by increasing the imidazole concentration. The fractions containing the eluted protein were pooled and concentrated for TEV cleavage, which was performed overnight at 4 °C. After the tag cleavage, proteins were further purified by gel filtration using a Superdex 75 increase 10/30 column (GE Healthcare) equilibrated with 50 mM Tris-HCl, 150 mM NaCl, and 2.5% (*v/v*) glycerol, pH 7.8. The purity of proteins was assessed by 18% SDS–PAGE analysis. The proteins were concentrated using a centrifugal filter (Merck Millipore) and the concentration was determined by UV absorbance using the corresponding ɛ values (M^−1^ cm^−1^).

### 4.4. CD Spectroscopy

CD measurements were obtained using a JASCO J-715 CD spectro-polarimeter equipped with a Peltier temperature controller (Model PTC-423S) at 4 °C in a 0.1 cm optical path length cell in the 200–260 nm wavelength range. Protein concentration was 0.2 mg/mL in 20 mM sodium phosphate buffer (pH 7.4). The data were recorded with a scanning speed of 20 nm/min and a band width of 1 nm. All spectra were averaged from three scans and baseline-corrected using a blank consisting of the protein buffer. The molar ellipticity per mean residue, [Ѳ] in deg·cm^2^·dmol^−^^1^, was calculated from the following equation: [Ѳ] = [Ѳ]obs × mrw × (10 × l × C)^−^^1^ where [Ѳ]obs is the ellipticity measured in degrees, mrw is the mean residue molecular mass, l is the optical path length of the cell in cm, and C is the protein concentration in g/L. During the melting experiments, CD spectra were collected with increasing temperatures, from 4 °C to 80 °C, with an average rate of 1 °C/min. Thermal denaturation was investigated by recording the CD signal at 222 nm. 

### 4.5. Light Scattering Measurements

Light scattering measurements were performed using a size exclusion chromatography (Superdex 75 increase 10/30, Cytiva, Italy) coupled with a light-scattering detector (miniDAWN TREOS, Wyatt Technology Corporation, Goleta, CA, USA) and a differential refractive index detector (Shodex RI-101, Wyatt Technology Corporation, Goleta, CA, USA). The measurements were obtained by loading ~1.0 mg of protein sample on the column equilibrated in 50 mM Tris–HCl, 150 mM NaCl, 2.5 (*v/v*)% glycerol (pH 7.8) as running buffer, at a flow rate of 0.5 mL/min. Data were recorded and analysed using the Astra software (version 5.3.4, Wyatt Technology Corporation, Goleta, CA, USA). 

### 4.6. Surface Plasmon Resonance

Assessments of binding kinetics and affinity between S-plugs with either the RBD or the S1 domain of the spike protein from SARS-CoV-2 were performed at 25 °C using Biacore T200 (Cytiva, Uppsala, Sweden). The spike RBD-His (SinoBiological, Cat: 40592-V08H) was stably captured at the surface of the CM5 sensor chip by means of an anti-histidine antibody (His Capture Kit, Cytiva) that had been covalently bound to the surface, as recommended by the manufacturer. In particular, the anti-histidine antibody provided in the His Capture Kit was diluted to 50 µg/mL in the immobilisation buffer included in the kit and covalently coupled to Sensor Chip CM5 by standard amine coupling to a level of approximately 12,000 RU. Then, the spike RBD-His was injected (25 μg·mL^−1^) over the anti-histidine antibody surface for 1 min. No protein was injected over the reference surface. The dissociation was monitored by injecting running buffer for 120 s. Surface regeneration was performed by injecting glycine buffer at a flow rate of 30 µL/min (10 mM, pH 1.5, 1 min).

The SARS-CoV-2 spike protein (S1) (Genscript, cat. Z03501-1) was immobilised on a CM5 sensorchip (98 µg/mL in 10 mM sodium acetate pH 4.59) at a flow rate of 10 µL/min using standard amine-coupling protocols to obtain densities of approximately 11000 RU. HBS-P+ buffer was used as a running buffer. All samples and buffers were degassed prior to use. For a positive control, ACE2-His protein (SinoBiological, Cat: 10108-H08H) was used.

Protein solutions (S-plug, S-plug2, S-plug3, ACE2) in HBS-P+ buffer at various concentrations were injected at 25 °C with a flow rate of 30 μL/min for 120 s (association phase), and then the buffer alone was injected for 600 s (dissociation phase). 

All mathematical manipulations and fitting operations were performed using BIAevaluation software (v2.02) provided with the Biacore T200 instrument (Cytiva) and assuming a 1:1 Langmuir binding model. 

## Figures and Tables

**Table 1:** Sequences of developed S-plugs. Most relevant residues for ACE2 interactions with spike RBD are reported in red.

	Sequence	Melting Temperature (Tm)
**S-plug**	STIEEQAKTFLDKFNHEAEDLFYQSSLASWNYNTNITEENVQNMNNAGDKWSAFLKEQSTLAQMYPLQEIQNQTVKQQLQALQQN	37 °C
**S-plug2**	SAMEEQAKFFLDKLQHELEDTQYQLLYKALALNKSQQEELQKQYKKTYDEYVTLAKKWAKAASSIPLNSITDEKLYKAMEKTASS	48 °C
**S-plug3**	SAMEEQAKFFLDKLQHELEDTQYQLLYKALALNKSQQEELQKQYKKTYDEYVTLAKKWAKAASSIPLNSITDEKLYKAMEKTASSPPGC	54 °C

**Table 2:** Binding parameters of SARS-CoV-2 spike to S-plugs and ACE2 receptor. Dissociation constants were measured by SPR.

Ligand	Analyte	K_D_ (nM)
SARS-CoV-2 S1	S-plug2	71.7 ± 1.8
RBD	S-plug2	61.7 ± 2.3
SARS-CoV-2 S1	S-plug3	31.7 ± 1.7
RBD	S-plug3	39.5 ± 1.1
RBD delta (B.1.617.2)	S-plug3	29.2 ± 0.6
SARS-CoV-2 S1	ACE2	51.9 ±1.4

## Data Availability

Plasmids are available on request from the corresponding author.

## Supplementary Materials

The following are available online at https://www.mdpi.com/article/10.3390/ijms23105601/s1.

## Abbreviations

COVID-19	Coronavirus disease 19
PDB	protein databank
RBD	receptor-binding domain
RBM	receptor-binding motif
ACE2	angiotensin-converting enzyme 2
SPR	surface plasmon resonance
