# Supplementary material for: Structure-Based Development of SARS-CoV-2 Spike Interactors
Source: Int J Mol Sci. 2022 May 17;23(10):5601. doi: 10.3390/ijms23105601 (PMC9144871; doi:10.3390/ijms23105601)
Supplement: Supplementary file 1 [file ijms-23-05601-s001.zip › ijms-1715097-supplementary.pdf]

# Supplementary Material

## Supplementary Tables

**Table S1.** MD structural model details

| MD1 | | Residues | # Res Atoms |
|---|---|---|---|
| | **S-plug** (ACE2-like)<br>Spike RBD domain | 19-103 (85 res.)<br>336-516 (181 res.) | 4174 |
| | Water molecules | 20108 | 60324 |
| | Ions ($Na^+$) | 2 | 2 |
| **MD2** | | | |
| | **S-plug2** (ACE2-like)<br>Spike RBD domain | 19-103 (85 res.)<br>336-516 (181 res.) | 4212 |
| | Water molecules | 20656 | 61968 |
| | Ions ($Cl^-$) | 2 | 2 |

**Table S2.** Spike–ACE2 binding affinities measured by different studies

| Protein coated/analyte | $K_D$ (nM) | Coating method | Detection method | References |
|---|---|---|---|---|
| SARS-CoV-2 Spike/ACE2 | 14.7 | Non-covalently immobilised to sensor chip | SPR | [1] |
| SARS-CoV-2 Spike/ACE2 | 1.2 | Non-covalently immobilised to sensor chip | Blitz | [2] |
| ACE2/SARS-CoV-2 RBD | 4.7 | Covalently immobilised to sensor chip | SPR | [3] |
| SARS-CoV-2 RBD/ACE2 | 44.2 | Covalently immobilised to sensor chip | SPR | [4] |

# Supplementary Figures

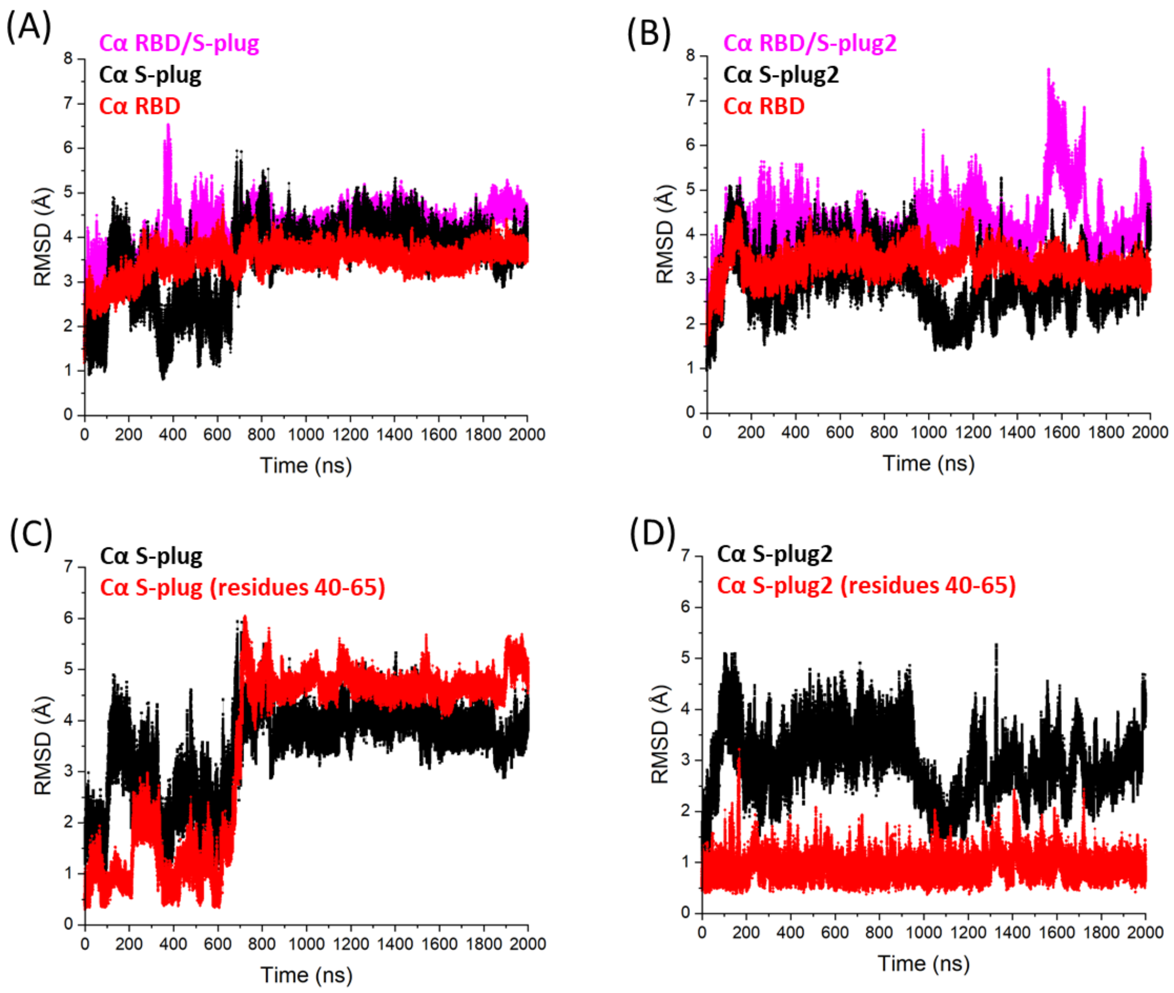


**Figure S1**. RMSD time evolution. (A) $C^{\alpha}$ RMSD values (MD simulation of S-plug/RBD) are reported in black, red and magenta for S-plug, RBD and the entire complex, respectively; **(B)** $C^{\alpha}$ RMSD values of (MD simulation of S-plug2/RBD) are reported in black, red and magenta for S-plug2, RBD and the entire complex, respectively; (C) $C^{\alpha}$ RMSD values computed for S-plug (black) and the region 40-65 $C^{\alpha}$ in the S-plug/RBD simulation; (D) $C^{\alpha}$ RMSD values computed for S-plug2 (black) and the region 40-65 $C^{\alpha}$ in the S-plug2/RBD simulation.

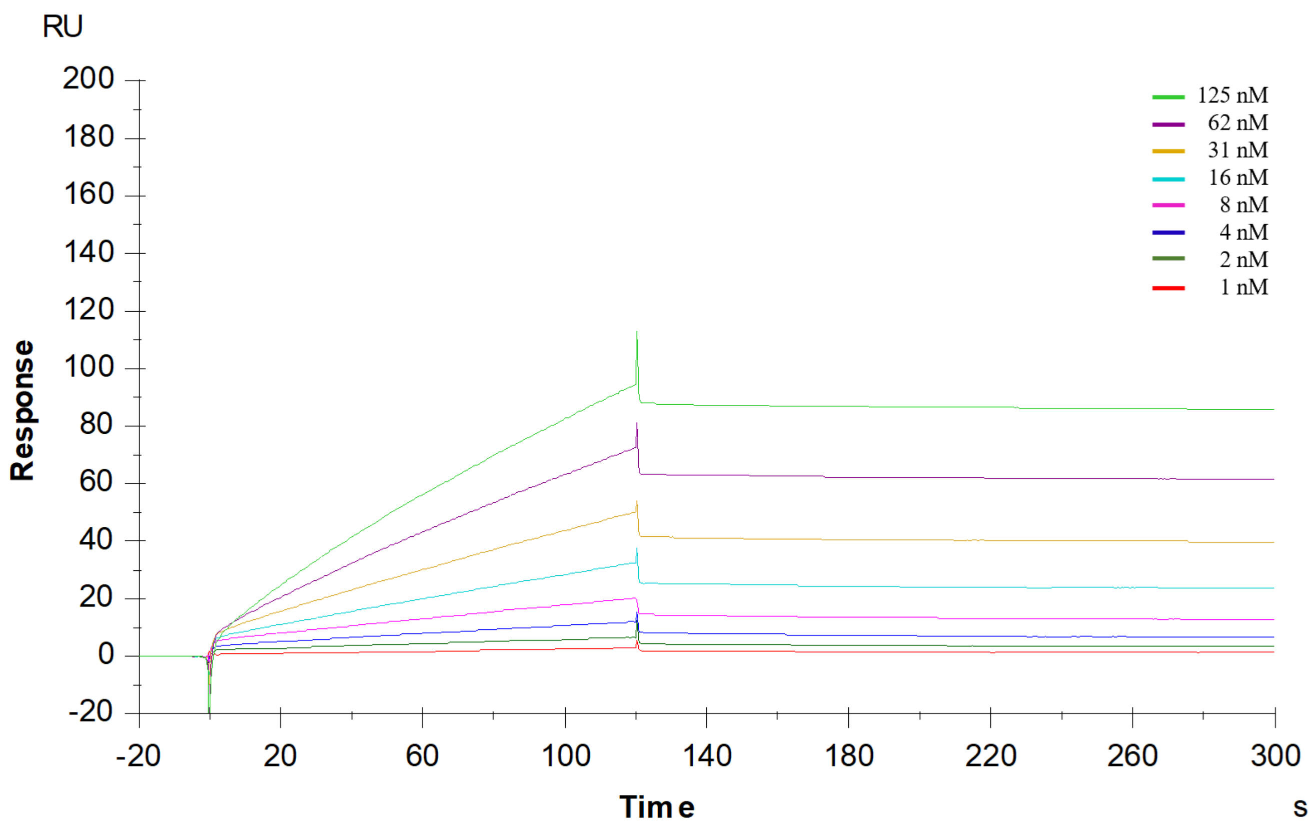


**Figure S2**. Surface Plasmon Resonance (SPR) analysis. Sensorgrams to evaluate the binding affinity of the host ACE2 domain to SARS-CoV-2 Spike S1 domain.

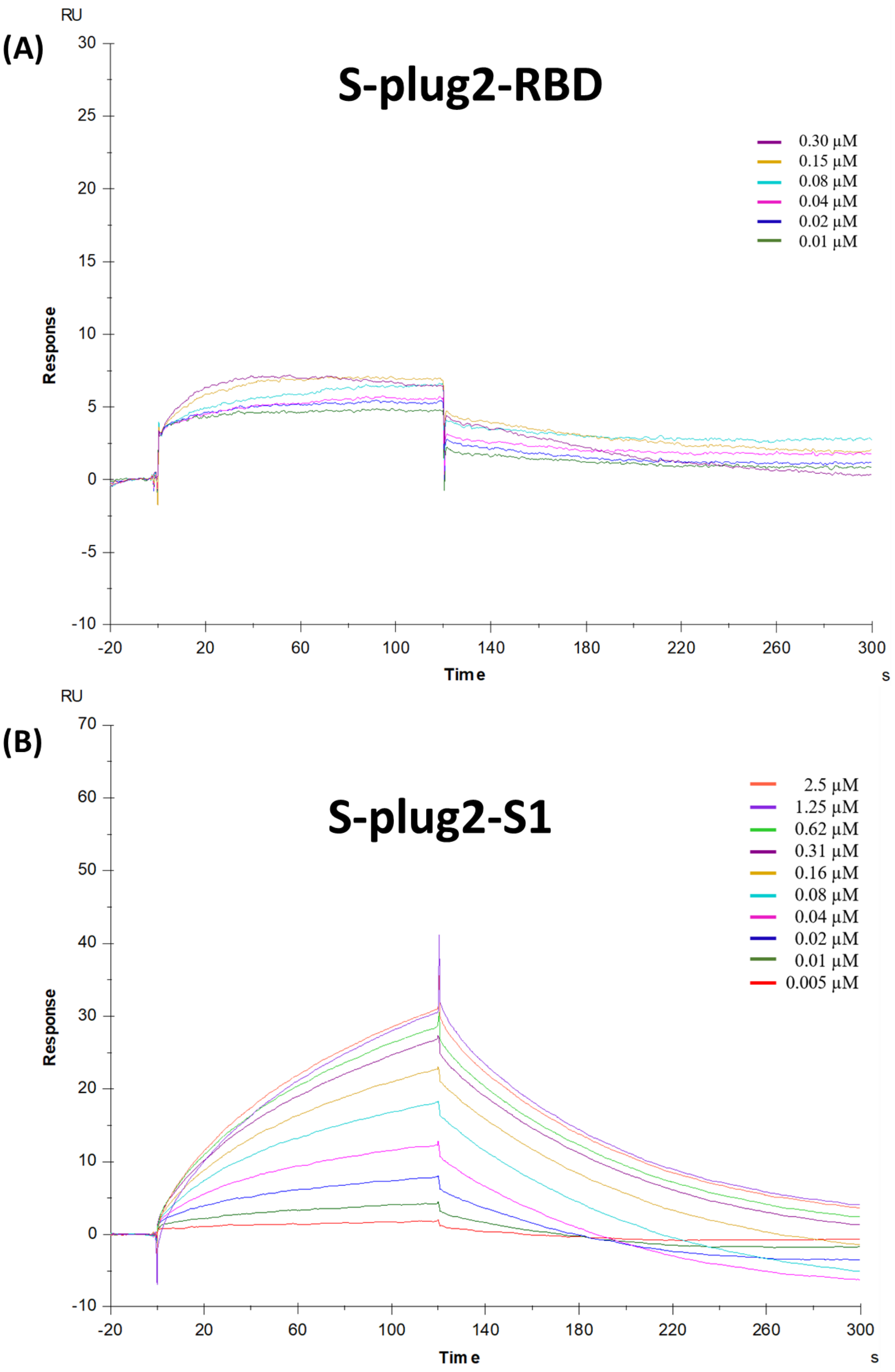


**Figure S3**. Surface Plasmon Resonance (SPR) analysis. Sensorgrams to evaluate the binding affinity of S-plug2 to (A) Spike RDB domain and (B) Spike S1 domain.

## Supplementary References


1. Wrapp, D.; Wang, N.; Corbett, K.S.; Goldsmith, J.A.; Hsieh, C.L.; Abiona, O.; Graham, B.S.; McLellan, J.S. Cryo-EM structure of the 2019-nCoV spike in the prefusion conformation. *Science* **2020**, *367*, 1260-1263, doi:10.1126/science.abb2507.

2. Walls, A.C.; Park, Y.J.; Tortorici, M.A.; Wall, A.; McGuire, A.T.; Veesler, D. Structure, Function, and Antigenicity of the SARS-CoV-2 Spike Glycoprotein. *Cell* **2020**, *181*, 281-292 e286, doi:10.1016/j.cell.2020.02.058.

3. Lan, J.; Ge, J.; Yu, J.; Shan, S.; Zhou, H.; Fan, S.; Zhang, Q.; Shi, X.; Wang, Q.; Zhang, L., et al. Structure of the SARS-CoV-2 spike receptor-binding domain bound to the ACE2 receptor. *Nature* **2020**, 10.1038/s41586-020-2180-5, doi:10.1038/s41586-020-2180-5.

4. Shang, J.; Ye, G.; Shi, K.; Wan, Y.; Luo, C.; Aihara, H.; Geng, Q.; Auerbach, A.; Li, F. Structural basis of receptor recognition by SARS-CoV-2. *Nature* **2020**, *581*, 221-224, doi:10.1038/s41586-020-2179-y.
